# Oxalate and oxalotrophy: an environmental perspective

**DOI:** 10.1093/sumbio/qvad004

**Published:** 2024-01-17

**Authors:** Don A Cowan, Darya Babenko, Ryan Bird, Alf Botha, Daniel O Breecker, Cathy E Clarke, Michele L Francis, Tim Gallagher, Pedro H Lebre, Teneille Nel, Alastair J Potts, Marla Trindade, Lonnie Van Zyl

**Affiliations:** Centre for Microbial Ecology and Genomics, Department of Biochemistry, Genetics and Microbiology, University of Pretoria, Pretoria 0002, South Africa; Department of Microbiology, University of Stellenbosch, Stellenbosch 7602, South Africa; Institute for Microbial Biotechnology and Metagenomics, Department of Biotechnology, University of the Western Cape, Cape Town 7535, South Africa; Department of Microbiology, University of Stellenbosch, Stellenbosch 7602, South Africa; Department of Earth and Planetary Science, the University of Texas at Austin, Austin, TX 78712-1692, United States; Department of Soil Science, University of Stellenbosch, Stellenbosch 7602, South Africa; Department of Soil Science, University of Stellenbosch, Stellenbosch 7602, South Africa; Department of Earth Sciences, Kent State University, Kent, OH 44240, United States; Centre for Microbial Ecology and Genomics, Department of Biochemistry, Genetics and Microbiology, University of Pretoria, Pretoria 0002, South Africa; Department of Soil Science, University of Stellenbosch, Stellenbosch 7602, South Africa; Botany Department, Nelson Mandela University, Gqeberha 6019, South Africa; Institute for Microbial Biotechnology and Metagenomics, Department of Biotechnology, University of the Western Cape, Cape Town 7535, South Africa; Institute for Microbial Biotechnology and Metagenomics, Department of Biotechnology, University of the Western Cape, Cape Town 7535, South Africa

**Keywords:** oxalate, oxalotrophy, oxalate-carbonate pathway (OCP), carbon sequestration, natural capital, nature based solutions

## Abstract

Oxalic acid is one of the most abundant organic acids produced by plants. Much of the global production of oxalic acid is deposited on soil surfaces in leaf litter to be oxidized by microorganisms, resulting in a pH increase and shifting the carbonate equilibria. In what is known as the oxalate-carbonate pathway, calcium oxalate metabolism results in CO_2_ being sequestered into soils as insoluble calcite (CaCO_3_). There is a growing appreciation that the global scale of this process is sufficiently large to be an important contribution to global carbon turnover budgets.

The microbiomics, genetics, and enzymology of oxalotrophy are all soundly established, although a more detailed understanding of the landscape-scale kinetics of the process would be needed to incorporate oxalotrophy as an element of process models informing the relevant Sustainable Development Goals. Here, we review the current state of knowledge of oxalotrophs and oxalotrophy and the role they play in terrestrial ecosystem services and functions in terms of carbon sequestration and nutrient cycling. We emphasize the relevance of these to the Sustainability Development Goals (SDGs) and highlight the importance of recognizing oxalotrophy, when accounting for the natural capital value of an ecosystem.

**Sustainability statement**

This review, Oxalate and oxalotrophy: an environmental perspective, addresses the impact of oxalate and oxalotrophy on the United Nations Sustainable Development Goals. Oxalates are common constituents of plant tissues. However, the environmental impact of these chemical constituents is principally derived from their decomposition by soil microorganisms, with the release of CO_2_ and stimulation of the oxalate-carbonate pathway and associated soil pH changes. These processes result in improved soil structure and quality with positive impacts on land and agriculture (Goals 2, 15), contribute directly to carbon sequestration (Goal 13), and contribute indirectly, through local and global effects, to other SDGs including Goal 3 (good health and wellbeing) and Goal 14 (life below water).

## Introduction

In the early 21st Century, we are witness to rates of biodiversity loss and ecosystem deterioration coupled with a rate of climate change that are unprecedented in human history (IPBES 2019, Fischer et al. 2021, Diffenbaugh et al. 2023). To combat a legacy of environmental destruction that is one of the defining characteristics of the Anthropocene (Folke et al. 2021), the Kunming-Montreal Global Biodiversity Framework was established in December 2022 at the 15th meeting of the Conference of Parties to the UN Convention on Biological Diversity (COP 15 2022). This framework identifies biodiversity as an important element in fighting climate change, poverty, and providing food security. The protection of biodiversity is understood to be critical in achieving many of the UN Sustainable Development Goals (SDGs) declared in the 2030 Agenda for Sustainable Development (UN General Assembly 2015), and remains an urgent priority (Sachs et al. 2023).

A vital future objective is the identification of ecosystems that contribute most to achievement of the SDGs and climate targets. The financial viability of Agriculture, Forestry, and Other Land Use (AFOLU) projects requires a cost analysis to be undertaken, based on the alternative land uses identified in the project's assessment (Verra 2019). A major issue with this approach is that ecosystem services are not accounted for, such that the true impact of a land use change cannot be valued or compared to alternatives. Without understanding the true value of ecosystem services, strategic decisions on ecosystem management, protection, or rejuvenation, designed to help achieve the SDGs (Sachs et al. 2023) may not necessarily be focused on the ecosystems that would offer the greatest return.

An ecosystem service that is not currently recognized for its value is oxalotrophy (the ability of organisms to use oxalate as a carbon and energy source: Kost et al. 2013). Microbial energy cycles that involve carbon-cycling are now recognized as being highly influential in the provision and regulation of ecosystem services. Oxalate salts, oxalic acid, and oxalogenic plants are widespread across the terrestrial world. The capacity for oxalotrophy, once thought to be a rare bacterial trait, is now believed to be widespread in prokaryotes and lower eukaryotes, and is found extensively in terrestrial ecosystems, plant-associated microbiomes and animal digestive tracts (Hervé et al. 2016).

The critical ecosystem service of oxalotrophy is related to the oxalate-carbonate pathway (Aragno and Verrecchia 2012, Palmieri et al. 2019), where CO_2_ is sequestered as insoluble carbonates in soils (Verrecchia 1990). In this pathway, oxalotrophic bacteria and fungi are able to decompose calcium oxalate in leaf litter and soil organic matter, forming CaCO_3_ in the process due to a local rise in pH (Pons et al. 2018, Uren 2018, Hervé et al. 2021). While there is a growing understanding of the scale and significance of this process, its importance in global ecosystem servicing is to date only poorly understood. Understanding the interactions between oxalogenic plants, saprophytic decomposers, and/or oxalotrophic microorganisms in pristine and agricultural soils is crucial to exploit this naturally occurring carbon capture process and possibly for the future engineering of carbon sequestration in drylands.

In this review and for the sake of brevity, we focus largely on oxalotrophic processes in terrestrial ecosystems. Related and equally important topics such as oxalogenesis (Palmieri et al. 2019) and the role of oxalate in a human clinical context (Galan-Llopis et al. 2023) have been recently and comprehensively reviewed.

## Oxalate salts and oxalic acid in the environment

Oxalic acid (C_2_H_2_O_4_) is one of the most common and important organic acids produced by plants and soil microorganisms in terrestrial ecosystems (Chen and Liao 2016). The salt of its conjugate base, oxalate, is also highly abundant, with calcium oxalate being the most common oxalate species in the environment (Gadd et al. 2014) and the most abundant organic biomineral found in terrestrial ecosystems (Baran and Monje 2008). Calcium oxalate includes mineral forms whewellite (monohydrate) and weddellite (dihydrate) (Verrecchia et al. 1993), collectively called CaOx hereafter. Other common oxalate salts found in plants and soils include magnesium oxalate (glushinskite) and highly soluble sodium and potassium oxalate species.

### Sources of oxalates

The two main sources of oxalates in terrestrial environments are plants and fungi (Cailleau et al. 2011, Martin et al. 2012, Parsons et al. 2022, Sonke and Trembath-Reichert 2023). There is little evidence of oxalogenesis in bacteria or archaea, with only a handful of reports on the process in specific Bacillota (previously Firmicutes): *Burkholderia* and *Pseudomonas genera* (Hamel et al. 1999, Nakata and He 2010, Nakata 2011). While more research on prokaryotic oxalogenesis is undoubtedly required, the current consensus is that plants and fungi are the primary contributors to environmental oxalate pools (Cailleau et al. 2011, Martin et al. 2012).

### Oxalates in plants

Oxalate/oxalic acid production in plants is common and serves several roles, including (i) the sequestration of excess Ca within plant tissues (Nakata 2003), (ii) the detoxification of phytotoxic metals both in the soil and in the plant (Ma et al. 2001), (iii) light regulation (Franceschi 2001), and (iv) protection against herbivory (Franceschi and Nakata 2005). Recently, the formation of CaOx has been linked to a drought adaptation in plants, providing a CO_2_ source during stomatal closure (Tooulakou et al. 2016). Such a drought adaptation would explain the strong negative correlation (r = −0.79) found between mean annual rainfall and percentage of plant species containing CaOx crystals (Karabourniotis et al. 2020) as well as the extreme accumulations of CaOx (*ca*. 100 kg in some cacti) in desert plants (Garvie 2006).

### Oxalates in soils

Oxalates are introduced into soils by root exudation, the microbial decomposition of plant litter, or via biosynthesis by soil fungi (Arnott 1995, Shimada et al. 1997, Jarosz-Wilkołazka and Gra̧z 2006, Verrecchia et al. 2006, Gadd et al. 2014, Hervé et al. 2021). Oxalates are often released by plants as a stress response in conditions of low nutrient availability or elevated levels of toxic metals (Adeleke et al. 2017). Acidic soils impose both the stress of low plant-available phosphorus (P) and phytotoxic aluminium (Al) concentrations. One of the key adaptation mechanisms for plants growing in acid soils is the production of oxalic acid either from the plant root itself or through the symbiotic relationship with mycorrhizal fungi (Dutton and Evans 1996; Ma et al. 1997, 2001; Gadd 2004, Fomina et al. 2005, Zhang et al. 2014). The chelation capacity of released oxalate reduces the phytotoxicity of Al while releasing phosphate sorbed to Fe and Al oxide surfaces. Oxalic acid production is also stimulated by ectomycorrhizal fungi in high pH environments where availability of P is reduced by interactions with calcite (Arvieu et al. 2003).

### Oxalates in fungi

Oxalic acid production by fungi is influenced by multiple environmental factors, such as available carbon and nitrogen sources, presence of metal oxalates, and pH (Gra̧z et al. 2009, Guggiari et al. 2011, Martin et al. 2012). Mycorrhizal and saprophytic fungi in soils are known to increase environmental oxalate reserves (Arvieu et al. 2003, Palmieri et al. 2019): these free-living, pathogenic, or plant symbiotic fungi either biosynthesize oxalic acid and excrete it into the environment or increase environmental oxalate levels by breaking down oxalate-rich plant matter and releasing the CaOx idioblasts into the surroundings (Gadd 2004; Verrecchia et al. 2006). In addition to nutrient provision and metal detoxification, the role of oxalic acid in fungi has been linked to pathogenicity (Williams et al. 2011, Vylkova 2017), competition (Gadd 2007, Lehner et al. 2008, Plassard et al. 2009), regulation of bacterial-fungal interactions (Deveau et al. 2018) and wood degradation (Shimada et al. 1997, Guggiari et al. 2011, Hatakka and Hammel 2011). CaOx crystals are closely associated with fungal hyphae (Uren 2018) and may be a by-product of ectomycorrhizal mineral weathering (Schmalenberger et al. 2015). These CaOx crystals, together with CaOx derived from decaying plant material, are the major sources of CaOx in soils (Uren 2018).

Despite the similar solubility of CaOx (K_sp_ = 2.7 × 10^−9^) and calcite (CaCO_3_; K_sp_ = 3.3 × 10^−9^) and the even greater solubility of gypsum (CaSO_4_·2H_2_O; K_sp_ = 3.1 × 10^−5^), the abundance of both calcite and gypsum in soils are much greater than that of CaOx (Uren 2018). The most common CaOx mineral, whewellite, is considered to be thermodynamically stable at ambient conditions (Verrecchia et al. 2006, Brecevic and Kralj 2010) and is not spontaneously oxidized. Thus, it is generally believed that the lower concentrations of CaOx in soils are predominantly related to the metabolic breakdown of CaOx by oxalotrophic microorganisms (Martin et al. 2012, Hervé et al. 2016). While oxalotrophy is likely to be the main factor controlling oxalate concentrations in soils, abiotic factors such as carboxylate sorption (Jones and Edwards 1998, Jones et al. 2003) and oxidation by manganese oxides (Stone 1987, Uren 2018) can also reduce the concentrations of extractable oxalates.

The high turnover rate and low concentrations of CaOx in most soils means it is not easily detected through mineralogical techniques like X-ray diffraction (XRD) (limit of detection 2%–5%). In addition, identification and quantification of oxalate species in soils is not straightforward (Adeleke et al. 2017). These combined factors may have led to the importance of CaOx in biogeochemical cycles being substantially underestimated until recently.

## Detection and quantitation of oxalate species

Techniques to detect and quantify oxalate salts and oxalotrophic activity are essential for an accurate understanding of the effects of oxalotrophy and oxalate dynamics at ecosystem scales. Accurate quantification of oxalate species in soil is challenging due to spatial heterogeneity and temporal variability of organic acids in soils (Jones et al. 2003, Adeleke et al. 2017). CaOx may be identified using a dye stain test, scanning electron microscopy (SEM), infrared (IR) spectroscopy, and XRD (Proia and Brinn 1985, Cailleau et al. 2011, Rojas-Molina et al. 2015, Schmitt et al. 2018, Francis and Poch 2019), but none of these methods are fully quantitative. Chemical analysis of oxalate-containing soils and vegetation typically involves extraction followed by quantitative analysis of oxalate in solution. Acid is used to dissolve sparingly soluble oxalate salts, and CaOx content may be determined by calculating the difference between total and water-soluble oxalate extracted from a sample (Ross et al. 1999, Savage et al. 2000, Xu et al. 2006, Tang et al. 2008, Savage and Mårtensson 2010, Liu et al. 2015, Schmitt et al. 2018). The strengths and limitations of various methods to quantify oxalates in solution, as well as details of limits of detection and quantification, linear ranges, and recovery efficiencies, have been recently reported (Misiewicz et al. 2023).

Enzymatic methods for quantitation of oxalate are based on detection of hydrogen peroxide produced by oxalate degradation using the enzyme oxalate oxidase. However, interference by other compounds in complex sample matrices is a serious limitation of this technique (Hansen et al. 1994, Zuo et al. 2010) and removal of reducing compounds and pH adjustment of samples is usually required (Keevil and Thornton 2006, Sigma-Aldrich Co. 2020). Some assay methods require addition of Ca^2+^ to promote oxalate precipitation (Certini et al. 2000, Cailleau et al. 2011, Mujinya et al. 2011, Naik et al. 2014, Liu et al. 2015, Shen et al. 2021), which adds uncertainty to the result if other Ca-bearing minerals are present and/or where Ca^2+^ ions need to be quantified.

Indicator methods for detection of oxalates in solution are based on alteration of fluorescence or ultraviolet (UV) radiation absorbance of indicator compounds, often containing copper, nickel, or iron (Holmes and Kennedy 2000, Chamjangali et al. 2006, Zhai et al. 2006, 2007, Tang et al. 2008, Zhai 2008, Chamjangali et al. 2009, Rhaman et al. 2014, Pourreza et al. 2018, Tavallali et al. 2018, Emami and Mousazadeh 2021, Hontz et al. 2020, Rocha et al. 2020, Shi et al. 2022, Chen et al. 2023). While these approaches are rapid and simple, most demonstrate interference effects by other organic anions. Electrochemical techniques, which are based on conductivity measurements (Holmes and Kennedy 2000, Strobel et al. 2004, Ardakani et al. 2006, Noblitt et al. 2009, Varão Moura et al. 2022, Kotani et al. 2023) are the most sensitive methods for the detection of oxalates in solution, but accuracy is affected by changes in bulk solution conductivity, and high oxalate concentrations cannot be measured accurately.

Liquid chromatography-mass spectroscopy (LC-MS) has shown high sensitivity, accuracy, and selectivity for oxalate analyses (Li et al. 2022, Mu et al. 2022, Misiewicz et al. 2023). However, the instrumentation and expertise required for this method is not necessarily available to all investigators and additional steps may still be necessary to prepare samples with complex matrices (Gómez et al. 2022). Other spectroscopic methods may also hold promise. Raman spectroscopy has been used to measure the ratio of whewellite to weddellite in kidney stones (Khalil and Azooz 2007). Semi-quantitative analysis of oxalic acid on clay surfaces has been achieved by IR spectroscopy (Zhang et al. 2018).

## Detection and quantitation of microbial oxalotrophy

Accurate methods for monitoring oxalate dynamics are essential in studies of the geochemical or biological processing of oxalate in the environment. The potential for oxalotrophy may be implied (but not proven) by the identification of oxalotrophic microorganisms and confirmation of their functional potential. Similarly, the potential capacity for oxalate metabolism can be identified by analysis of the key genes encoding enzymes of the oxalate degradation pathways (*oxc, frc, oxdC, and oxlT*: Sonke and Trembath-Reichert 2023), either by gene-specific PCR or via full genome or metagenome sequencing. While the presence of a single gene in a multi-gene pathway is not, in itself, evidence of active metabolism, the presence of a complete pathway is more definitive, particularly if supported by transcriptomic data (i.e. evidence of active gene expression). The contribution of both fungi and bacteria to oxalate degradation has been confirmed by qPCR analysis of the *frc* gene (Martin et al. 2012).

Quantitation of oxalate degradation provides definitive evidence of oxalotrophy, and has been monitored qualitatively by observation of the dissolution of oxalate salts around growing microbial colonies (Braissant et al. 2002, 2004). Oxalotrophic bacterial activity has also been monitored by isothermal microcalorimetry (Bravo et al. 2011). Oxalate degradation in microbial cultures may be monitored quantitatively by measuring depletion of oxalate in the medium by chemical analytical methods (Ström et al. 2001, van Hees et al. 2002, Daniel et al. 2007, Fujii et al. 2013, Dauer and Perakis 2014). This allows determination of oxalate mineralization rates (Ström et al. 2001, van Hees et al. 2002, Van Hees et al. 2005).

Alkalinity is arguably the most easily-measured fermentation parameter but is completely nonselective. Bicarbonate ions are produced as a by-product of oxalate oxidation and thus an increase in solution pH may be considered, under very specific fermentation conditions, as confirmation of oxalotrophic activity (Braissant et al. 2002, 2004; Cailleau et al. 2011, 2014; Martin et al. 2012, Rowley et al. 2017, Gatz-Miller et al. 2022).

Mineralogical and petrographic studies have proved useful in the identification of oxalate transformation processes and in constructing biogeochemical models of oxalate processing pathways (Verrecchia and Dumont 1996, Cailleau et al. 2011). Mineral substrates and products of oxalotrophy may be identified and micromorphologically described by use of techniques such as X-ray fluorescence microscopy (XFM), XRD, SEM, and optical microscopy (Braissant et al. 2004, Garvie 2006, Cailleau et al. 2011, Bonazza et al. 2015, Francis and Poch 2019, Parsons et al. 2022).

A novel approach for detecting and perhaps quantifying oxalotrophy involves determination of the respiratory quotient (RQ). RQ is the number of moles of CO_2_ produced per mole of O_2_ consumed during respiration (Dilly 2001). This quotient is, in part, controlled by the oxidation state of carbon in the substrate. The oxidation state of carbon is much higher in oxalate (+3) than in carbohydrates (0), making the RQ for oxalate oxidation (4) much larger than the RQ of aerobic respiration involving carbohydrate oxidation (1) and most other organic substrates (+0.7–+1.3) (Masiello et al. 2008). Therefore, determinations of RQ from measurements of CO_2_ and O_2_ concentrations in the headspace of incubation vessels and the air-filled pore spaces of soils (e.g. Gallagher and Breecker 2020, Hicks Pries et al. 2020), although not yet to our knowledge applied for this purpose, may enable nondestructive monitoring of oxalotrophy.

## The biochemistry and genetics of oxalotrophy

### Genes, pathways, and enzymes

**Figure 1.**
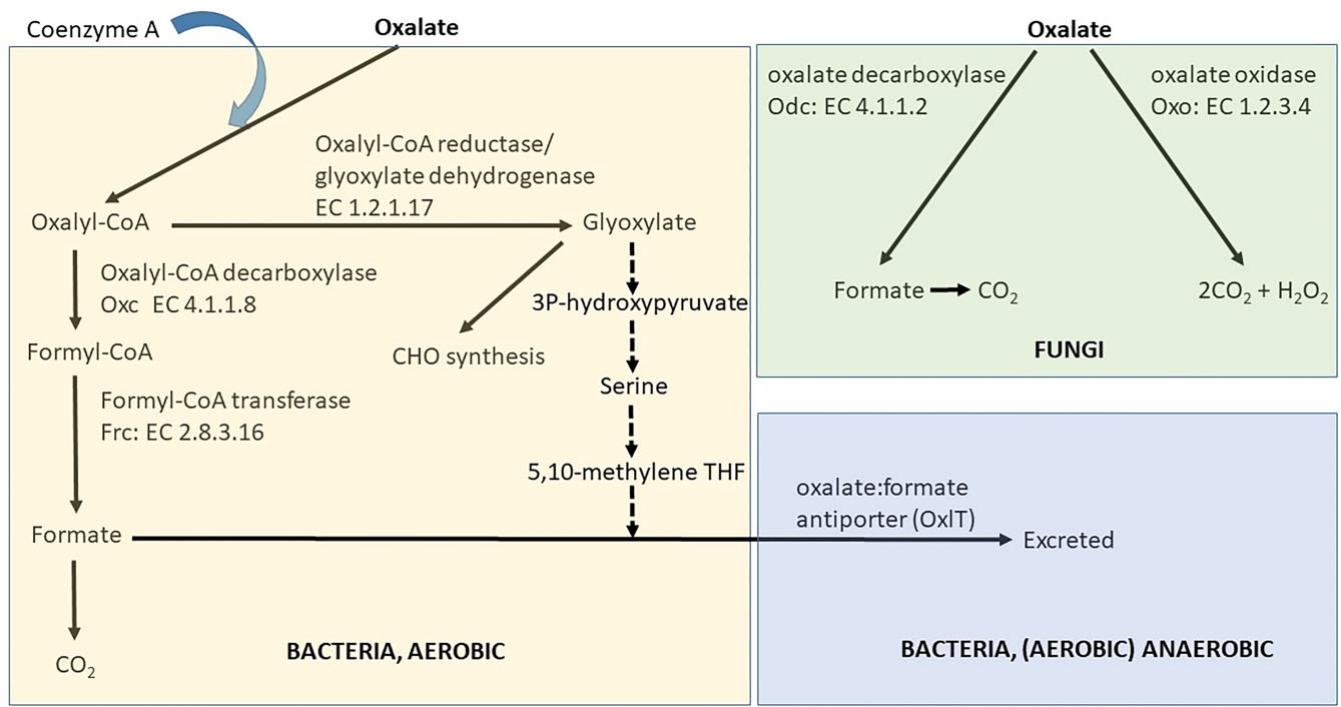
Diagrammatic summary of the principal oxalotrophic pathways, intermediates and enzymes (adapted from Palmieri et al. 2019, Robertson and Meyers 2022, Sonke and Trembath-Reichert 2023).

The capacity for oxalotrophy is widespread in both prokaryotes and lower eukaryotes, and can occur both aerobically and anaerobically (Fig. 1). In bacteria, aerobic oxidation of oxalate occurs via a CoA-dependent activation to oxalyl-CoA, which is either decarboxylated by oxalyl-CoA decarboxylase [Oxc: EC 4.1.1.8] with subsequent formyl-CoA transferase (Frc: EC2.8.3.16]-catalyzed CoA removal to generate formate (for subsequent oxidation to CO_2_) or converted to glyoxylate by oxalyl-CoA reductase [glyoxylate dehydrogenase: EC 1.2.1.17] for subsequent carbohydrate synthesis via the glycine/serine pathways or energy production via the TCA cycle. Under anaerobic conditions, the primary oxidation product, formate, is excreted from the cell via an oxalate: formate antiporter (OxlT), with the associated H^+^-dependent membrane polarization driving APT synthase activity.

An alternative pathway for aerobic oxalate catabolism, where oxalate is indirectly converted to formate via the glyocolate pathway and the use of a C1 tetrahydrofolate intermediate, has been recently proposed (Robertson and Meyers 2022). Formate may be oxidised to CO_2_ with the synthesis of NADH or excreted via an oxalate-formate antiporter, hitherto only implicated in anaerobic oxalate metabolism. This pathway (Fig. 1), which may be specific to the actinobacterial genus *Kribella*, does not contain the first accepted oxalotrophic marker enzyme gene (*oxc*). Although the gene *frc* is present in some of the oxalotrophic *Kribbella* species, it is not thought to play any role in the proposed oxalotrophy pathway for this genus.

In fungi and other lower eukaryotes, oxalate is oxidized by two enzymes: oxalate decarboxylase [OdcC: EC 4.1.1.2] and oxalate oxidase [Oxo: EC 1.2.3.4]. The detailed enzymology of these pathways has been comprehensively reviewed by Palmieri et al. (2019).

The key genes for oxalotrophy (*oxc, frc, odc*, and *oxo*), together with the oxalate-formate antiporter gene (*oxlT*) are widely used as markers for identification of (putative) oxalotrophic microbial taxa (Sonke and Trembath-Reichert 2023). Such approaches have the potential to identify both novel oxalotrophic taxa and novel oxalotrophic environments, although the discovery that alternative pathways (Robertson and Meyers 2022), which do not use the oxalyl-CoA decarboxylase/formyl-CoA transferase pathway highlights the weaknesses of marker-specific functional surveys. Furthermore, enzyme substrate-promiscuity implicates other enzymes in fulfilling the role of the oxalyl-CoA reductase, demonstrated in *Kribella* spp. (Robertson and Meyers 2022) and in *Methylorubrum extorquens* (Scheider et al. 2012). It is therefore clear that estimations of the diversity and capacity of oxalotrophy will inevitably be underestimated using *oxc* and *frc* surveys.

A recent multilocus survey of multiple metagenome sequence datasets, including high quality Metagenome Assembled Genomes (MAGs), suggested that microbial oxalotrophy is widespread in marine habitats, and may be an important trophic strategy in marine sediments (Sonke and Trembath-Reichert 2023).

The genetic basis of oxalotrophy (i.e. gene organisation, gene copies and the effect of biotic and abiotic factors on the regulation of expression) has not been widely investigated and has only been described for a few bacterial models. For example, the *oxc* and *frc* genes have been shown to be transcribed as an operon in lactic acid bacteria and in *E. coli* (Federici et al. 2004, Azcarate-Peril et al. 2006, Lewanika et al. 2007). A more recent study by Jiang et al. (2020) showed that the two marker genes form an operon in a broad range of species, although some operons also include a third gene encoding a transporter protein (e.g. in *Escherichia coli* G3/10) or a CoA: oxalate CoA-transferase (e.g. in *Acetobacter pasteurianus*). By contrast, in *Oxalobacter formigenes* the genes have independent promoters and are located several kilobases apart on the genome (Sidhu et al. 1997).

The regulation of expression of these marker genes has also been understudied, although environmental pH has been identified as an important regulator of microbial oxalate gene expression. For instance, in *Bifidobacterium animalis oxc* gene expression is upregulated by a pH increase from 4.5 to 6.5 (Turroni et al. 2010), whereas in *Lactobacillus acidophilus* this increase occurred between pHs 4.5 to 5.5 but was reduced at a pH of 6.8 (Azcarate-Peril et al. 2006).

## Microbiology of oxalotrophs

### Bacterial oxalotrophy

Culture-dependent studies of oxalotrophy have underpinned our understanding of the microbiology and biochemistry of the oxalotrophic process. Some oxalotrophic bacteria are completely dependent on oxalic acid as a carbon source and energy (“specialist” oxalotrophs), whereas others can utilize oxalic acid in addition to other carbon sources (“generalist” oxalotrophs) (Sahin 2003). Based on culture-dependent studies, the capacity for bacterial oxalotrophy is not distributed across all taxa, and was initially considered as a rare trait attributed to cultured representatives of the *Actinomycetota* (previously *Actinobacteria*), *Bacillota*, and *Pseudomonadota* (previously *Proteobacteria*) phyla, predominantly from terrestrial ecosystems (e.g. soil, freshwater sediments, plants, gut habitats, and other host-associations) (Table 1) (Hervé et al. 2016, Robertson and Meyers 2022). The recent application of metagenomic analyses has extended this capacity to the *Bacteroidota* (previously *Bacteriodetes*) (Tanca et al. 2017) and to marine environments (Sonke and Trembath-Reichert 2023), highlighting the likelihood that many more taxa may contribute to oxalotrophic carbon cycling than generally thought.

**Table 1.** Inventory of cultured oxalotrophic bacteria and fungi and methods used to detect and measure oxalotrophic activity.

Taxa	Source	Oxalotrophy detection method	Oxalate utilization	References
Bacteria				
***Pseudomonadota***				
***Achromobacter* spp**.	Soil	Degradation halo on media with CaOx as the sole C-source		Bravo et al. 2015
***Acinetobacter baylyi*** ***A. calcoaceticus*** ***Acinetobacter*** **sp**.	Leaves	Degradation halo on media with KOx as the sole C-source		Ghate et al. 2021
***Afipia carboxidovorans***	Soil	Growth with Ox as the sole C-source		Paul et al. 2010
***Afipia* sp**.	Soil	Degradation halo on media with CaOx as the sole C-source		Bravo et al. 2015
***Agrobacterium bv.1***	Tropical legumes	Positive oxalate assimilation test		De Lajudie et al. 1994
***Agrobacterium* sp**.	Soil, rhizospheres, plant stem tissue	Degradation halo on media with CaOx as the sole C-source	0.209 µmol/h, Isothermal microcalorimetry (IMC)	Sahin et al. 2002, Bravo et al. 2015
***Aminobacter carboxidus***	Soil	Growth with CaOx as the sole C-source		Jenni et al. 1988
***Ancylobacter* sp**.	Soil, rhizospheres, plant stem tissue	Degradation halo with CaOx as the sole C-source		Sahin et al. 2002
***Ancylobacter* spp**.	Soil	Growth with KOx as the sole C-source		Lang et al. 2008
***Azospirillum brasilense***	Tropical plants	Growth in Oxalate-enrichment culture		Weber et al. 1999, Sahin et al. 2002
***Azorhizobium oxalatiphilum***	Plant	Growth in Oxalate-enrichment culture		Lang et al. 2013
***Beijerinckia indica***	Soil	Growth with oxalate as the sole C-source		Becking 1984, Tamas et al. 2010
***Bradyrhizobium japonicum***	Legume root nodules	Degradation of CaOx/NaOx in liquid media as the sole C-source		Koch et al. 2014
***Burkholderia* sp**.	Soil, rhizospheres, plant stem tissue	Degradation halo with CaOx as the sole C-source		Sahin et al. 2002
***Burkholderia spp*.**	Surface of *Varroa destructor*	Growth with CaOx as the sole C-source		Maddaloni and Pascual 2015
***Burkholderia spp*.**	Plant	Degradation halo on media with CaOx as the sole C-source		Kost et al. 2014
***Cupriavidus* sp**.	Soil	Degradation halo on media with CaOx as sole C-source		Bravo et al. 2015
***Cupriavidus oxalaticus***	Soil, intestinal tract of Indian earthworm	IMC measurements in liquid media with KOx	0.66 µmol/h, IMC	Khambata and Bhat 1953; Bravo et al. 2013a, b
***Cupriavidus necator***	Soil, Leaf litter	IMC measurements in liquid media with KOx	0.40 µmol/h, IMC	Braissant et al. 2002; Bravo et al. 2013a, b
***Desulfovibrio vulgaris***	Sediment	Growth with NaOx as sole C-source		Postgate 1963
***Enterobacter* spp**.	Leaves	Degradation halo with KOx as sole C-source		Ghate et al. 2021
***Ensifer meliloti***	Legumes	Activity increase with increasing oxalate concentration, IMC		Baaziz et al. 2021
***Ensifer* sp**.	Soil	Degradation halo on media with CaOx as sole C-source		Bravo et al. 2015
***Herminiimonas glaciei***	Soil	Growth with oxalate as sole C-source		Sahin et al. 2010
***Herminiimonas saxobsidens***	Lichen-colonized rock	Growth with KOx as sole C-source		Lang et al. 2007
***Hyphomicrobium vulgare***	River water	Growth with oxalate as sole C-source		Urakami and Komagata 1987
***Janthinobacterium* spp**.	Subtropical stream	Growth with NaOx as sole C-source		Lu et al. 2020
***Lysobacter soli***	Soil	Degradation halo on media with CaOx as sole C-source	0.221 µmol/h, IMC	Bravo et al. 2015
***Lysobacter* spp**.	Soil	Degradation halo on media with CaOx as sole C-source		Bravo et al. 2015
***Mesorhizobium* sp**.	Legumes	Growth with Ox as sole C-source		Tan et al. 1999
***Methylorubrum extorquens***	Soil	Growth in Oxalate-enrichment culture	0.32 µmol/h, IMC	Bassalik and Bassalik 1913, Sahin et al. 2002, Bravo 2013
***Oxalicibacterium flavum***	Leaf litter	Growth with KOx as sole C-source		Tamer et al. 2002
***Oxalicibacterium solurbis***	Soil	Growth with oxalate as sole C-source		Sahin et al. 2010
***Oxalobacter formigenes***	Mammalian gastrointestinal tract	Degradation halo with CaOx as sole C-source	2.9 mmol/min/g cell (dry weight)	Allison et al. 1985, Daniel et al. 2021
***Oxalobacter vibrioformis***	Freshwater sediment	Growth with di-ammonium oxalate as sole C-source		Dehning and Schink 1989
***Pandoraea* sp**.	Leaf litter	Growth with CaOx as sole C-source		Martin et al. 2012
***Paracoccus alcaliphilus***	Rhizoplane	Degradation halo around colonies on media with KOx as sole C-source		Anbazhagan et al. 2007
***Pectobacterium carotovorum***	Leaves	Degradation halo with KOx as sole C-source		Ghate et al. 2021
***Polaromonas* sp**.	Soil	Degradation halo on media with CaOx as sole C-source		Bravo et al. 2015
***Providencia rettgeri***	Oxalate consumption	5 g/L consumed in 48 h, HPCE		Hokama et al. 2005
***Pseudomonas* sp**.	Soil	[^14^C]-labeled CO_2_ respired from dual-labeled [^14^C]-oxalic acid	0.0090 µmol/g biomass in 48 h	Morris and Allen 1994
***Pseudomonas* sp**.	Leaves	Degradation halo with KOx as sole C-source		Ghate et al. 2021
***Pseudoxanthomonas* sp**.	Soil	Degradation halo on media with CaOx as sole C-source		Bravo et al. 2015
***Rhizobium huakuii***	Root nodules	Positive oxalate assimilation test		De Lajudie et al. 1994
***Rhodospirillum* sp**.	Sediments	Growth with Ox as sole C-source		Saleem and Rehman 2019
***Sphingomonas* sp**.	Soil	Degradation halo with CaOx as sole C-source		Bravo et al. 2013a, b
***Starkeya novella***	Soil	Growth studies in liquid media		Starkey 1935, Chandra and Shetna. 1977
***Stenotrophomonas maltophilia***	Soil	Degradation halo on media with CaOx as sole C-source	0.166 μmol/h, IMC	Bravo et al. 2015
***Stenotrophomonas* sp**.	Soil	Degradation halo on media with CaOx as sole C-source	0.074 μmol/h, IMC	Bravo et al. 2015
***Stenotrophomonas* spp**.	Soil	Degradation halo on media with CaOx as sole C-source		Bravo et al. 2015
***Variovorax paradoxus***	Leaf litter	Degradation halo around colonies on media with Ox as the C-source		Tamer and Aragno 1980, Martin et al. 2012
***Variovorax soli***	Soil	Degradation halo on media with CaOx as sole C-source	0.240 µmol/h, IMC	Bravo et al. 2015
***Variovorax* sp**.	Soil	Degradation halo on media with CaOx as sole C-source	0.186 µmol/h, IMC	Bravo et al. 2015
***Xanthobacter autotrophicus***	Leaf litter	Growth in CaOx enriched liquid media	Rapid consumption of oxalate	Braissant et al. 2002
***Xanthobacter* sp**.	Soil	Degradation halo with CaOx as sole C-source		Sahin et al. 2002
***Xanthomonas* spp**.	Soil	Degradation halo on media with CaOx as sole C-source	0.164 µmol/h, IMC	Bravo et al. 2015
***Bacillota***				
***Ammoniphilus oxalaticus***	Rhizosphere of *Rumex acetosa* and decaying wood	Growth with NH_4_Ox as sole C-source		Zaitsev et al. 1998
***Ammoniphilus oxalativorans***	Rhizosphere of *Rumex acetosa* and decaying wood	Growth with NH_4_Ox as sole C-source		Zaitsev et al. 1998
***Bacillus licheniformis***	Soil	[^14^C]-labeled CO_2_ respired from dual-labeled [^14^C]-oxalic acid	0.0039 µmol/g biomass per 48	Morris and Allen 1994
***Bacillus* sp**.	Soil	Degradation halo on media with CaOx as sole C-source	0.024 µmol/h, IMC	Bravo et al. 2015
***Clostridium sporogenes***	Gastrointestinal tract of the white-throated woodrat	Oxalate degradation assays	18.4%–40.3% removal in 48 h	Miller et al. 2014
***Cohnella phaseoli***	Soil	Degradation halo on media with CaOx as sole C-source		Bravo et al. 2013b
***Enterococcus gallinarum***	Gastrointestinal tract of the white-throated woodrat	Oxalate degradation assays	18.4%–35.5% removal in 48 h	Miller et al. 2014
***Enterococcus faecium***	Dog faeces	Oxalate degradation, IMC	13.2% after 72 h, IMC	Ren et al. 2011
***Enterococcus faecalis***	Dog faeces/human stool	Oxalate degradation, IMC	25.3% after 72 h, IMC	Hokama et al. 2000, Ren et al. 2011
***Lactiplantibacillus plantarum***	Fermented foods and human stool	Oxalate degradation in liquid media	1.4% oxalate degradation in 32 h, Enzymatic assay	Campieri et al. 2001
***Lacticaseibacillus casei***	Dairy products	Oxalate degradation in liquid media	>90% oxalate degraded in 5–6 days, Enzymatic assay	Kwak et al. 2006
***Lactobacillus gasseri AM63T***	Human intestine	Oxalate degradation in liquid media	74% oxalate degraded in 5 days, Enzymatic assay	Lewanika et al. 2007
***Lactobacillus gasseri***	Gastrointestinal tract of the white-throated woodrat	Oxalate degradation assays	14.5%–24.8% removal in 48 h	Miller et al. 2014
***Lactobacillus gasseri*.**	Functional foods; pharmaceutical preparations	Oxalate degradation in liquid media	35%–100% oxalate degraded in 5 days, Enzymatic assay	Turroni et al. 2007
***Lactobacillus johnsonii***	Gastrointestinal tract of the white-throated woodrat	Oxalate degradation assays	29%–36% removal in 48 h	Miller et al. 2014
***Lactobacillus plantarum***	Functional foods; pharmaceutical preparations	Oxalate degradation in liquid media	20%–40% oxalate degraded in 5 days, Enzymatic assay	Turroni et al. 2007
***Lactobacillus rhamnosus***	Functional foods; pharmaceutical preparations	Oxalate degradation in liquid media	20%–47% oxalate degraded in 5 days, Enzymatic assay	Turroni et al. 2007
***L. acidophilus***	Functional foods; pharmaceutical preparations	Oxalate degradation in liquid media	35–100% oxalate degraded in 5 days, Enzymatic assay	Turroni et al. 2007
***L. acidophilus***	Human stool	Oxalate degradation in liquid media	11.79% oxalate degradation in 25 h, Enzymatic assay	Campieri et al. 2001
***L. acidophilus***	Human stool	Oxalate degradation in liquid media	24% oxalate degraded in 40 h; Enzymatic assay	Azcarate-Peril et al. 2006
***Lactobacillus salviarius***	Functional foods; pharmaceutical preparations	Oxalate degradation in liquid media	20% oxalate degraded in 5 days, Enzymatic assay	Turroni et al. 2007
***Lactococcus garvieae***	Dog faeces	Oxalate degradation, IMC	15.7% removal in 72 h, IMC	Ren et al. 2011
***Levilactobacillus brevis***	Dairy products	Oxalate degradation in liquid media	0.94% oxalate degradation in 27 h, Enzymatic assay	Campieri et al. 2001
***Leuconostoc lactis***	Dog faeces	Oxalate degradation, IMC	21.8% after 72 h, IMC	Kwak et al. 2006, Ren et al. 2011
***Leuconostoc mesenteroides***	Dog faeces	Oxalate degradation, IMC	14.7% removal in 72 h, IMC	Weese et al. 2004, Ren et al. 2011
***Ligilactobacillus animalis***	Gastrointestinal tract of the white-throated woodrat	Oxalate degradation assays	27.6% removal in 48 h	Miller et al. 2014
***Moorella thermoacetica***	Horse faeces	Growth studies in oxalate-containing media	94% oxalate degradation in 200 h, HPLC	Daniel and Drake 1993
***Oxalophagus oxalicus***	Freshwater sediments	Growth with di-ammonium oxalate as sole C-source		Collins et al. 1994, Dehning and Schink. 1989
***Paenibacillus* sp**.	Soil	Degradation halo on media with CaOx as sole C-source	0.117 μmol/h, IMC	Bravo et al. 2015
***Psychrobacillus* spp**.	Soil	Degradation halo on media with CaOx as sole C-source		Bravo et al. 2015
***Streptococcus salivarius subsp. thermophilus***	Milk	Oxalate degradation in liquid media	3.06% oxalate degradation in 2.5 h, Enzymatic assay	Campieri et al. 2001
***Actinomycetota***				
***Arthrobacter* sp**.	Soil	Degradation halo on media with CaOx as sole C-source		Bravo et al. 2015
***Bifidobacterium animalis***	Human stool/animal faeces	Oxalate degradation in liquid media	100% oxalate used after 120 h	Turroni et al. 2010
***Bifidobacterium longum subsp. infantis***	Gastrointestinal tract of human infants	Oxalate degradation in liquid media	5.26% oxalate degradation in 36 h, Enzymatic assay	Campieri et al. 2001
***Bifidiobactium spp*.**	Animal/human faeces, probiotic products	Sodium oxalate degradation in liquid media	Oxalate-degrading capacity ranging from 1–60%, Enzymatic assay	Federici et al. 2004
***Curtobacterium* sp**.	Surface of *Varroa destructor*	Growth with CaOx as sole C-source		Maddaloni and Pascual 2015
***Eggerthella lenta***	Human stools	Oxalate degradation in liquid media	1 mg/ml of oxalate removed in 24 h, HPLC	Ito et al. 1996
***Kribbella spp*.**	Soil	Degradation halo with CaOx or NaOx as sole C-source		Robertson and Meyers 2022
***Lentzea albidocapillata***	Soil	Growth studies in oxalate-containing media		Lee et al. 2000, Xie et al. 2002
***Lentzea aerocolonigenes***	Soil	Growth studies in oxalate-containing media		Xie et al. 2002
***Mycobacterium smegmatis***	Soil/Freshwater	Growth on malachite green oxalate		Gordon and Mihm 1959; Wayne & Kubica 1986
***Nocardia* sp**.	Soil	[^14^C]-labeled CO_2_ respired from dual-labeled [^14^C]-oxalic acid	0.0011 µmol/g biomass per 48 h	Muller 1950, Morris and Allen 1994
***Saccharothrix spp*.**	Soil	Growth with Ox as sole C-source		Labeda et al. 1992
***Streptomyces achromogenes***	Soil	Degradation halo on media with CaOx as the C-source	0.150 ± 0.009 μmol/h, IMC	Bravo et al. 2015
***Streptomyces violaceoruber***	Soil	IMC measurements in liquid media with KOx	0.29 µmol/h, IMC	Bravo et al. 2011
***Streptomyces* sp**.	Soil	Degradation halo with CaOx as sole C-source	0.26 µmol/h, IMC	Knutson et al. 1980, Bravo et al. 2015
***Terrabacter* sp**.	Soil	Degradation halo with CaOx as sole C-source		Bravo et al. 2013a, b
**Fungi**				
***Ascomycota***				
***Aspergillus niger***	Culture collection	CO_2_ release from oxalic acid as sole C source	2.3–12.8 μmoles CO_2_/g dry mycelial/h	Emiliani and Bekes 1964
***Bartalinia* sp**.	Leaf litter	Growth with CaOx as sole C-source		Hervé et al. 2021
***Fusarium chlamydosporum***	Soil	Growth with CaOx as sole C-source		Simon et al. 2016
***Fusarium equiseti***	Soil	Growth with CaOx as sole C-source		Simon et al. 2016
***Fusarium nygamai***	Soil	Growth with CaOx as sole C-source		Simon et al. 2016
***Fusarium oxysporum***	Soil	Growth with CaOx as sole C-source		Simon et al. 2016
***Fusarium* sp**.	Leaf litter	Growth with CaOx as sole C source		Hervé et al. 2021
***Myrothecium verrucaria***	Plant	CO_2_ release from oxalic acid as sole C source	16.6—19.6 μmoles CO_2_/20 μmoles oxalic acid	Lillehoj and Smith 1965
***Nigrosabulum* sp**.	Leaf litter	Growth with CaOx as sole C-source		Hervé et al. 2021
***Ochroconis* sp**.	Leaf litter	Growth with CaOx as sole C-source		Hervé et al. 2021
***Pseudocoleophoma* sp**.	Leaf litter	Growth with CaOx as sole C-source		Hervé et al. 2021
***Purpureocillium* sp**.	Leaf litter	Growth with CaOx as sole C-source		Hervé et al. 2021
***Sclerotinia sclerotiorum* B24 and SS41**	Culture collection	Photometric measurements with oxalic acid as sole C source	12.2–25.8 μg oxalate decarboxylated/min/mg protein	Magro et al. 1988
***Sclerotinia sclerotiorum* WT ‘1980’**	Plant	Odc activity measured by quantifying formate produced via FDH-coupled assay	16 μmole formate produced/mg tissue in 20 min	Liang et al. 2015
***Sirastachys* spp**.	Leaf litter	Growth with CaOx as sole C-source		Hervé et al. 2021
***Trichoderma* sp**.	Leaf litter	Growth with CaOx as sole C-source		Hervé et al. 2021
***Basidiomycota***				
***Abortiporus biennis***	Culture collection	Oxo activity determined by the formation of H_2_O_2_	1.6–6.4 U/mg	Gra̧z et al. 2009
***Agaricus bisporus***	Soil and plant litter	ODC activity determind by formate production; HPLC	65–111 U/mg protein	Kathiara et al. 2000
***Agaricus blazei***	Wood-associated	Growth and dissolution with CaOx as sole C-source		Guggiari et al. 2011
***Agrocybe aegerita***	Wood-associated	Growth and dissolution with CaOx as sole C-source		Guggiari et al. 2011
***Armillaria japonica***	Wood-associated	CO_2_ release from oxalic acid as sole C source		Shimazono 1955
***Armillaria mellea***	Wood-associated	CO_2_ release from oxalic acid as sole C source		Shimazono 1955
***Ceriporiopsis subvermispora***	Wood-associated	Formate production with oxalate as sole C source for Odc activity; H_2_O_2_ production for Oxo activity.	Odc: 0.081 nkat/mg protein Oxo: 0.07–0.08 nkat/mg protein	Watanabe et al. 2005
***Ceriporiopsis subvermispora* FP-10572**	Wood-associated	Oxo activity assay measured H_2_O_2_ released during oxalic acid oxidation	K_M_: 0.1 mM *k_cat_*: 88 s^−1^	Aguilar et al. 1999
***Collyvia veltipes***	Wood-associated	CO_2_ release from oxalic acid as sole C source	840–1440 CO_2_ evolved mm^3^/30 mins over 40 d	Shimazono 1955
***Coriolus hirsutus***	Wood-associated	CO_2_ release from oxalic acid as sole C source	27–38 CO_2_ evolved mm^3^/30 mins over 40 d	Shimazono 1955
***Coriolus versicolor***	Wood-associated	CO_2_ release from oxalic acid as sole C source		Shimazono 1955
***Dichomitus squalens* PO114**	Wood-associated	Odc activity using enzyme kit in oxalic acid induced broth	86 μmol oxalic acid decarboxylated/mg protein/min	Mäkelä et al. 2002
***Flammulina (Collybia) velutipes***	Wood-associated	Measured liberated ^14^CO_2_ from ^14^C-oxalic acid solution in the presence of citrate	166 μmol/min/mg protein	Mehta and Datta 1991
***Flammulina velutipes***	Wood-associated	HPX 87-H column used to measure OA decrease in plant tissue transformed with with *oxd* gene		Kesarwani et al. 2000
***Flammulina velutipes***	Wood-associated	Measured liberated ^14^CO_2_ from ^14^C-oxalic acid solution in the presence of citrate		Chakraborty et al. 2013
***Flammulina velutipes***	Wood-associated	Growth with CaOx as sole C-source		Guggiari et al. 2011
***Flammulina velutipes* ATCC13547**	Wood-associated	Induced Odc expression with oxalic acid		Kamthan et al. 2015
***Flammulina velutipes* IJF 140502**	Wood-associated			Dias et al. 2006
***Fomitopsis rhodophaea***	Wood-associated	CO_2_ release from oxalic acid as sole C source		Shimazono 1955
***Ganoderma tsugae***	Wood-associated	Growth with CaOx as the sole C-source		Guggiari et al. 2011
***Laetiporus sulphureus***	Wood-associated	Growth with CaOx as the sole C-source		Guggiari et al. 2011
***Lenzites betulina***	Wood-associated	CO_2_ release from oxalic acid as sole C source		Shimazono 1955
***Lyophyllum ulmarium***	Wood-associated	Growth with CaOx as the sole C-source		Guggiari et al. 2011
***Phanerochaete chrysosporium* BKM-F-1767 (ATCC 24725)**	Grape tissue	Aa sequence identified using LCMS		Sato et al. 2007
***Pharnerochaete sanguinea* T51**	Wood-associated	ODC activity using enzyme kit	15 μmol oxalic acid decarboxylated/mg protein^/^min	Mäkelä et al. 2002
***Phellinus Hartigi***	Wood-associated	CO_2_ release from oxalic acid as sole C source		Shimazono 1955
***Pholiota adiposa***	Wood-associated	CO_2_ release from oxalic acid as sole C source		Shimazono 1955
***Pholiota mutabilis***	Wood-associated	CO_2_ release from oxalic acid as sole C source		Shimazono 1955
***Pholiota nameko***	Wood-associated	Growth with CaOx as sole C-source		Guggiari et al. 2011
***Pleurotus citrinopileatus***	Wood-associated	Dissolution of oxalate crystals in solid medium		Guggiari et al. 2011
***Pleurotus eryngii***	Wood-associated	Growth with CaOx as sole C-source		Guggiari et al. 2011
***Pleurotus ostreatus***	Wood-associated	Growth with CaOx as sole C-source		Guggiari et al. 2011
***Pleurotus tuberregium***	Wood-associated	Growth with CaOx as sole C-source		Guggiari et al. 2011
***Polyporus ciliatus***	Wood-associated	Growth with CaOx as sole C-source		Guggiari et al. 2011
***Postia placenta* MAD698**	Wood-associated	Spectrophotometric test kit for oxalate anion release	0.42–0.51 units/mg protein	Micales 1997
***Pycnoporus cinnabarinus***	Wood-associated	Oxalate consumption by HPLC		Guggiari et al. 2011
***Shizophillum commune***	Wood-associated	CO_2_ release from oxalic acid as sole C source		Shimazono 1955
***Tilletia contraversa***	Wood-associated	Oxalic acid oxidation measured by respiratory ^14^CO_2_ from ^14^C-oxalate		Vaisey et al. 1961
***Trametes ochracea* T7**	Wood-associated	ODC activity using enzyme kit	9 μmol oxalic acid decarboxylated/mg protein^/^min	Mäkelä et al. 2002
***Trametes sanginea***	Wood-associated	CO_2_ release from oxalic acid as sole C source		Shimazono 1955
***Trametes suaveolens***	Wood-associated	Dissolution of crystals on solid medium		Guggiari et al. 2011
***Trametes versicolor* PRL572**	Wood-associated	Oxd activity by photometric measurement of CO_2_ from oxalic acid as sole C source	1–2.25 U/mg protein	Zhu and Hong 2010
***Trametes versicolor* R/7**	Wood-associated	Odc activity using enzyme kit	41 μmol oxalic acid decarboxylated/mg protein/min	Mäkelä et al. 2002
***Deuteromycota***				
***Coniothyrium minitans* ZS-1**	Plant-associated	Oxalate degradation measured by HPLC		Xu et al. 2022
***Mucormycota***				
***Absidia* spp**.	Leaf litter	Growth with CaOx as sole C-source		Hervé et al. 2021
***Lichtheimia* sp**.	Leaf litter	Growth with CaOx as sole C-source		Hervé et al. 2021

Due to the critical role that bacteria play in oxalate-carbonate pathway processes in natural ecosystems, the detection, identification, and characterization of these microorganisms are essential for understanding their contribution to ecosystem functioning. Although cultrable bacteria only represent a small percentage of the total population, culture-based studies provide critical, and unique datasets, such as process kinetics and interorganismal interactions, that contribute to our understanding of the role of microorganisms in the oxalate-carbonate pathway. For example, cultivation studies have led to the suggestion that certain *Insecta* hosts actively recruit bacteria capable of degrading oxalic acid in order to minimize oxalate toxicity (Kikuchi et al. 2005, Compant et al. 2008, Itoh et al. 2014, Maddaloni and Pascual 2015, Tago et al. 2015). Similarly, plant rhizosphere function has been shown to be highly dependent on *Burkholderia* spp. oxalotrophy (Kost et al. 2014).

Culture-dependent studies of oxalotrophs have primarily focused on enumeration and taxonomy, particularly via the use of selective growth media (Table 1), generally employing solid media containing CaOx as a sole carbon source (Khammar et al. 2009, Bravo et al. 2011, Cailleau et al. 2014, Sun et al. 2019). Potassium, sodium, and ammonium oxalate salts have also been successful in culturing oxalotrophs, and are consumed at similar rates to CaOx (Tamer and Aragno 1980, Tamer 1982, Campieri et al. 2001, Sahin et al. 2002, Bravo et al. 2015, Robertson and Meyers 2022). *Kribbella* spp. are an exception, being inhibited by sodium oxalate but capable of metabolising CaOx very rapidly (Robertson and Meyers 2022). Despite the use of traditional cultivation techniques being limited to only a handful of media, the diversity of culturable oxalotrophic bacteria is steadily increasing (Table 1) and new species are expected to be found when targeting new environments (Bravo et al. 2015, Sonke and Trembath-Reichert 2023) and/or with refinements in culture media and culturing technologies (Wu et al. 2020).

Almost all growth kinetics and oxalate consumption characterization have been conducted on a few model bacterial strains (Table 1) and very few studies have assessed the ecological role of cultivable oxalotrophic bacteria. Due to the high oxidation state of oxalate, bacterial growth rates on oxalate as a sole carbon source are generally low (Braissant et al. 2002), and the relationship between bacterial growth and oxalate degradation is species-dependent (Campieri et al. 2001, Bravo et al. 2015). An apparent decoupling between cell growth and oxalate consumption suggests that not all strains use oxalate solely for biomass production.

The highest oxalate consumption rates for soil oxalotrophs are recorded for *Pseudomonadota* species. *Cupriavidus oxalaticus* (generalist) and *C. necator* (specialist) consumed calcium and potassium oxalate at rates greater than 0.6 µM h^−1^ and 0.4 µM h^−1^, respectively (Bravo et al. 2011). *Variovorax soli* C18 consumed potassium oxalate at a rate of 0.24 µM h^−1^ (Bravo et al. 2015). Members of the *Actinomycetota* also demonstrated comparable consumption rates (up to 0.29 µM h^−1^) (Bravo et al. 2011, 2015).

### Fungal oxalotrophy

A dynamic relationship exists between fungi, oxalic acid, and the environment (Gadd 1999, Gadd et al. 2014, Palmieri et al. 2019). Much of the work on oxalate processing by fungi has focused on oxalogenesis rather than oxalotrophy, a process dominated by basidiomycetous brown and white wood-rot fungi from soil environments (Shimada et al. 1997, Munir et al. 2001, Mäkelä et al. 2002, Jarosz-Wilkołazka and Gra̧z 2006, Guggiari et al. 2011). Filamentous Asco- and Mucoromycetes have also been shown to be capable of oxalic acid biosynthesis. Some fungal taxa, such as *Aspergillus, Fusarium*, and *Pleurotus* spp. are capable of both oxalogenesis and oxalotrophy, switching from anabolism to a catabolic metabolism under nutrient-rich conditions. The duality in fungal oxalate metabolism may explain why oxalotrophy in the environment has largely been overlooked in lower eukaryotes (Gadd 1999, Guggiari et al. 2011, Palmieri et al. 2019).

The enzyme mostly reported to be associated with fungal oxalotrophy is oxalate decarboxylase (OXD: EC 4.1.1.2), which degrades oxalic acid into formate and carbon dioxide (CO_2_) (Shimazono 1955, Kathiara et al. 2000, Mäkelä et al. 2002, Zhu and Hong 2010, Kamthan et al. 2015, Kumar et al. 2016). This mainly intracellular enzyme, which is also found in bacteria (Just et al. 2004), occurs within vesicles close to the plasma membrane, as well as attached to the fungal cell wall (Dutton et al. 1994, Micales 1997, Kathiara et al. 2000, Sato et al. 2007, Mäkelä et al. 2009). It was predicted that OXD occurs in most lignocellulose-degrading basidiomycetes, but it is best documented for *Flammulina* (*Collybia*) *velutipes* (Mehta and Datta 1991, Kesarwani et al. 2000, Dias et al. 2006, Guggiari et al. 2011, Chakraborty et al. 2013, Kamthan et al. 2015), *Trametes* (*Coriolus*) *versicolor* (Shimazono 1955, Dutton et al. 1994, Mäkelä et al. 2002, Zhu and Hong 2010, Guggiari et al. 2011), and *Pholiota* spp. (Guggiari et al. 2011).

A second enzyme found to be associated with fungal oxalotrophy is oxalate oxidase (OXO, oxalate: oxygen oxidoreductase, EC 1.2.3.4) (Aguilar et al. 1999, Svedružić et al. 2005, Watanabe et al. 2005, Gra̧z et al. 2009). This enzyme, which has been reported in the white-rot fungi *A. bienni* and *C. subvermispora*, cleaves oxalate into two CO_2_ molecules and hydrogen peroxide (Aguilar et al. 1999, Gra̧z et al. 2009). Both OXD and OXO activities have been reported for *C. subvermispora*, (Mäkelä et al. 2009). The presence of multiple genes associated with oxalotrophy in a single genome suggests that different enzyme isoforms may play different physiological roles in oxalotrophic fungi (Mäkelä et al. 2010).

Formate dehydrogenase (FDH, EC 1.2.1.2. and 1.2.2.1) may also play an important role in fungal oxalotrophy, since this enzyme can convert potentially toxic formate (produced by OXD) to CO_2_ (Watanabe et al. 2005). The co-location of the *fdh* and *oxd* genes in fungal genomes (Sato et al. 2009) also suggests a functional link between the two gene products, a concept supported by observations of the co-expression of *odc* and *fdh* genes in some basidiomycetes (Sato et al. 2007, 2009).

Although the genes implicated in oxalotrophy have been recorded in many fungi, relatively little is known of their gene expression control, the detailed structures and functional properties of enzymes, or their physiological roles. The potential link between oxalic acid degradation and energy generation is also poorly understood.

Analogous to the role of oxalic acid in fungus-plant symbioses (Dutton et al. 1996), it is suggested that oxalic acid may play important role in bacterial-fungal interactions (Deveau et al. 2018). Simon et al. (2016) proposed that oxalotrophic bacteria in soil environments are generally nonmotile and require fungal hyphae as a ‘transit’ mechanism to locate environmental oxalate reserves (Bravo et al. 2013a). It has been demonstrated that fungus-derived oxalic acid can act as a chemoattractant for motile oxalotrophic bacteria, which then utilize the secreted oxalic acid as a carbon and energy source (Haq et al. 2018, Rudnick et al. 2015).

The role of fungi in oxalate-carbonate pathway processes is poorly understood, although it is generally appreciated that the contribution of oxalotrophic fungi is under-estimated (Martin et al. 2012, Hervé et al. 2021).

## Environmental microbiomics of oxalotrophs

### Phylogenetic diversity of oxalotrophic taxa

Recent advances in molecular techniques, particularly metabarcoding and metagenomic methods, have allowed researchers to investigate the uncultured fraction of oxalotrophic taxa in virtually any environment and at scales from local to regional to global.

Specific marker genes are widely accepted as indicators of the capacity for oxalotrophy in different groups of organisms and under different environmental conditions (*oxdC*, bacterial, anaerobic; *oxc, frc, oxlT*, bacterial, aerobic; *odc, oxo*, fungal: see Fig. 1). Nevertheless, caution is advised in generating functional interpretations from gene marker data. The *frc* gene is not unique to oxalotrophy and is not considered to be adequate as a sole molecular marker for this process (Khammar et al. 2009). The inclusion of both *oxdC* and *oxc* genes as markers is, however, considered to be sufficient to confirm the presence of microbial oxalotrophy (Sonke and Trembath-Reichert 2023). Alternatively, it has been suggested that the entire oxalate operon, with both the *oxc* and *frc* genes, is a valid marker for oxalotrophy (Azcarate-Peril et al. 2006).

In fungi, the genes encoding the oxalate decarboxylase (EC 4.1.1.2) or oxalate oxidase (EC 1.2.3.4) (Grąz et al. 2023) (see Fig. 1) could be used as markers, although, to the authors’ knowledge, the latter has not been used in any molecular marker studies to date.

Notwithstanding the power of eDNA-based methods, metabarcoding approaches rely on conserved consensus sequences and therefore may not identify novel or highly sequence-variable oxalotrophic genes (Simon et al. 2016).

Alternative approaches to functional prediction using phylogenetic datasets, such as the “function from phylogeny” algorithms PiCRUSt (https://picrust.github.io/picrust) and Tax4Fun (http://tax4fun.gobics.de), could also aid in the identification of oxalotrophic capacity. These programs generate functional predictions based on alignments between metabarcoding data and genomic data from known genomes (Douglas et al. 2020, Wemheuer et al. 2020), and are therefore solely predictive in nature. However, they do provide a valuable basis for further experimental verification. A study of termite gut microbiomes (Suryavanshi et al. 2016) demonstrated that the use of PICRUSt could predict oxalotrophic functional genes via KEGG Orthology. This study inferred the presence of a highly diverse oxalotrophic community in the termite gut, suggesting the presence of five different oxalotrophic pathways with formate, carbon dioxide or hydrogen peroxide as potential metabolic end-products.

A pioneering study (Sahin 2003) suggested that most oxalotrophic taxa identified in public sequence databases were aerobes and clustered into either proteobacterial or actinobacterial clades. Gram-negative oxalotrophic bacteria belonged to various Pseudomonadota subclasses (α, β, and γ with one δ), whereas Gram-positive oxalotrophs aligned with the Bacillota and Actinomycetota phyla.

Probably the most comprehensive phylogenetic analysis of the key oxalotrophy genes (*oxc, frc, oxdC*, and *oxlT)* was a recently published (Sonke and Trembath-Reichert 2023) bioinformatic study of publicly available genome and metagenome datasets. *Oxc* genes clustered into three primary Groups, each including several separate clades: Group I included multiple genera from the phylum Actinomycetota, Group II consisted of several Pseudomonadota-dominated clades largely of terrestrial origin, while Group III was composed exclusively of Alphaproteobacteria but from diverse origins, including hydrothermal vent waters, cold marine sediments, plant microbiomes, and human gut samples.

O*xdC* genes clustered into two primary groups, each of multiple clades. As for *oxc* gene phylogeny, taxonomic assignments within clades were mostly homogeneous [Pseudomonadota and Bacillota for prokaryotes, and various fungal phyla and the chlorophyte Chloroflexota (previously Choroflexi)]. The homogeneous clustering of genes according to taxonomic groups was interpreted as evidence for vertical inheritance rather than Horizontal Gene Transfer (although some evidence for the latter was noted) (Sonke and Trembath-Reichert 2023).

Similar phylogenetic distributions have been observed using other marker gene sets. Khammar et al. (2009) used degenerate primers to identify both *frc* and *oxc* genes. The study revealed a high taxonomic similarity between *frc* genes, most of which grouped within α- and β-proteobacterial clades. The β-proteobacterial cluster included *frc* genes positioned adjacent to *oxc* genes, thought to be indicative of an oxalate operon (Azcarate-Peril et al. 2006).

A comparison of the cultured oxalotroph diversity with putative oxalotrophic taxa identified in sequence datasets suggests that a high proportion of oxalotrophs remain uncultured (e.g. Barka et al. 2016). Given that a proportion of marker sequences cannot be assigned to any known taxa (Sun et al. 2019), it is also possible that these “dark” oxalotrophs may harbor alternative oxalotrophic pathways (Janssen 2006).

**Figure 2.**
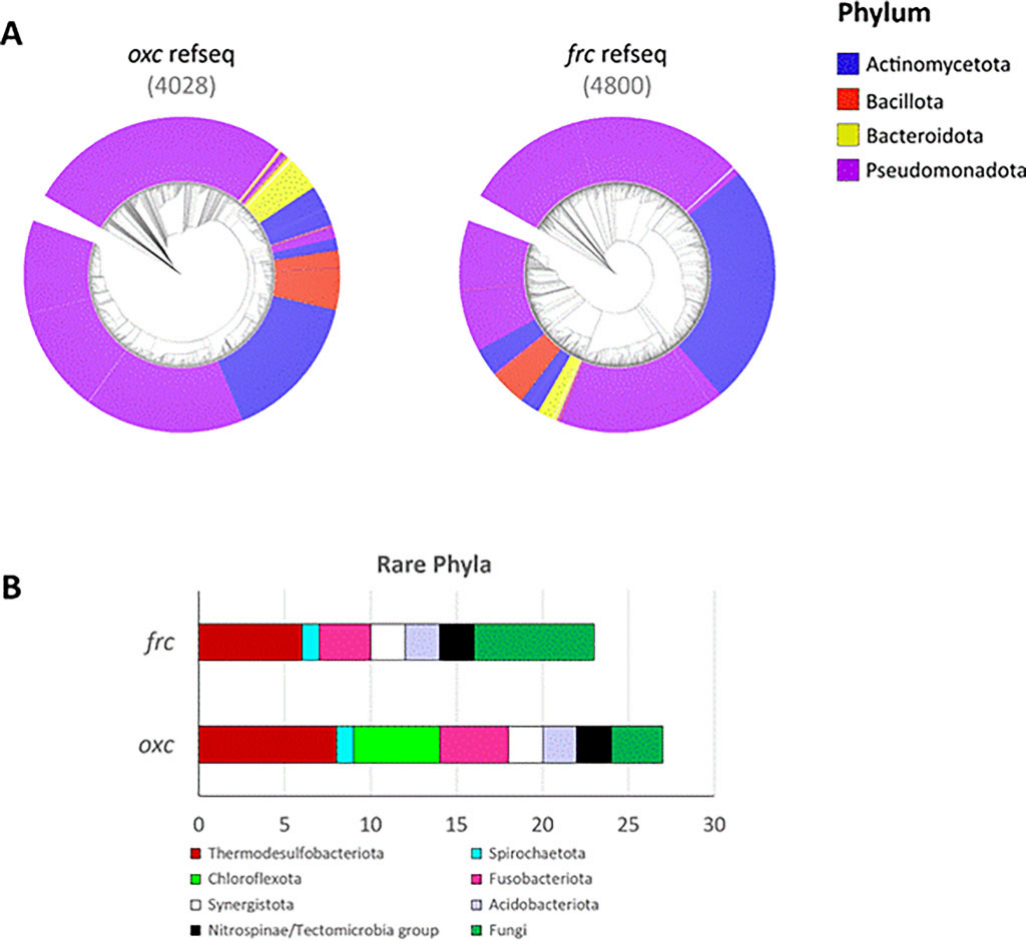
(A) Phylogenetic trees of the protein sequences of the two essential oxalotrophic genes, oxalyl-CoA decarboxylase (OXC) and formyl-CoA transferase (FRC). Sequences were extracted from the Refseq protein database. Numbers of sequences used to draw the trees are shown in brackets above each tree. The sequences were first aligned using MAFFT and the tree calculated using FastTree v2.0, while the trees were drawn using the Itol webserver. Sequences belonging to the dominant phyla (phyla that contained >1% of the total number of sequences for each marker gene) are highlighted in the tree using color strips. (B) Barplot showing the number of OXC and FRC sequences, retrieved from the RefSeq database belonging to rare phyla (i.e. phyla that contained <1% of total retrieved sequences for either marker gene).

An up-to-date phylogenetic survey of OXC and FRC protein sequences from the NCBI RefSeq database (Fig. 2) shows, as previously reported (Palmieri et al. 2019), that these oxalotrophic markers are mainly present in four phyla: Pseudomonadota, Bacillota, Bacteroidota (previously Bacteriodetes), and Actinomycetota (Fig. 2A). In the case of OXC, the sequences from Bacteroidota, Actinomycetota and Bacillota form a phylogenetic cluster distinct from the majority of Pseudomonadota sequences, suggesting that they are more closely related. By contrast, the phylogenetic clustering of the FRC proteins is less evident, with the Bacillota and Bacteroidota sequences clustering together in a distinct phylogenetic clade. The sequences for both markers were also found in taxa that are not often associated with oxalotrophy (Fig. 2B). These include the Fusobacteriota genus *Cetobacterium*, which is often associated with fish intestinal tracts (Ramirez et al. 2018, Qi et al. 2023), and the bacterial marine sponge symbiont *Entotheonella palauensis* (from the Nitrospinae/Tectomicrobia group) (Lackner et al. 2017, Schmidt et al. 2000). The Chloroflexi genus *Tepidiforma* was found to only contain sequences for the *oxc* gene, and a recent study (Palmer et al. 2023) has shown that two members in this genus, *Tepidiforma flava* and *Tepidiforma thermophila*, were unable to grow with oxalate as a sole carbon source, further suggesting that both marker genes are essential for oxalotrophy. While both OXC and FRC proteins were found in Fungi, none of the fungal taxa in the database contained both markers, consistent with the view that fungi catabolize oxalate using alternative enzyme pathways (Fig. 1).

Phylogeography of oxalotrophic taxa: Although best known for their presence (and function) in soil ecosystems (Palmieri et al. 2019), plant rhizospheres (Voronina and Sidorova 2017) and the human gut (Sadaf et al. 2017), oxalotrophs now appear to be present in widely diverse habitats, including oxic and anoxic, mesophilic and thermophilic, oligotrophic and copiotrophic, halophilic and nonhalophilic and neutrophilic and acidophilic environments. Metagenomic sequence data suggest that oxalotrophic capacity, encoded by both prokaryotes and lower eukaryotes and probably associated with multiple taxa in any specific habitat, exists in environments as diverse as soils, compost, plant-associated communities (e.g. rhizospheric and ectomycorrhizal microbiomes), waste and fresh water, acid mine drainage, marine water, deep sea hydrothermal fluids, marine sediments, and animal and invertebrate gut systems (Suryavanshi et al. 2016, Tanca et al. 2017, Sonke and Trembath-Reichert 2023).

## Ecosystem services and functions of oxalotrophy in relation to the SDGs

### Sustainable agriculture (SDG 2: promote sustainable agriculture)

Oxalotrophic bacteria have been studied in numerous natural ecosystems, but their presence and role in agricultural soils and their association with crops is not well understood. Oxalotrophy and oxalate production are important in recruiting plant-beneficial endophytic bacteria for important crops such as lupin and maize (Kost et al. 2013). Oxalotrophy has also been highlighted as a possible way to ameliorate acid soils (Xu et al. 2019). Soil acidity affects 40% of arable land and is a major problem affecting food security in many developing countries where lime sources are unaffordable. As a result, alternative ways to buffer acid soils are highly sought after. Xu et al. (2019) examined the effect of CaOx addition to acid soils fertilized either by manure or NPK fertilizer. Their findings demonstrated that oxalotrophy in both fertilized and manured soils increased soil pH, but manured soils responded more rapidly to CaOx addition due to functional redundancy. While the application of CaOx to agricultural soils is not economically feasible, the potential of oxalotrophy to reduce soil acidity should be further explored, especially given how prevalent CaOx can be in crop residues.

Oxalotrophy can also play a role in the soil stability of cultivated fields. In a study on the CaOx content of soybean plants (*Glycine max*), it was suggested that the rapid breakdown of CaOx contained in unharvested crop residues is important for Ca recycling. This rapid release of Ca was suggested to maintain aggregate stability and good physical soil structure in the intensively cultivated Mollisols of Argentina (Borrelli et al. 2016). More work to understand the role of oxalotrophy and Ca release in other crop residues is important, as this could be a further advantage of minimum tillage practices, which aim to retain plant residues in the soil.

### Nutrient cycling in ecosystems (SD 15: Life on land)

The role of CaOx in ecosystem Ca cycling is deemed to be an important issue (Dauer and Perakis 2014, Turpault et al. 2019, Parsons et al. 2022) with the view that Ca dynamics are different from other basic cation cycles due to fungal CaOx production and subsequent microbial breakdown resulting in rapid Ca turnover (Dauer and Perakis 2014). Calcium is an essential nutrient in plants and animals and understanding the cycling of Ca in the environment is essential for sustainable management of ecosystems and their biodiversity.

Calcium is often a limiting nutrient in weathered tropical ecosystems and dystrophic forest soils (Dauer and Perakis 2014, Turpault et al. 2019). A large component (23%–40%) of the total Ca in detritus of temperate forest ecosystems is made up of CaOx (Dauer and Perakis 2014). This biomineral and its microbial breakdown is increasingly being recognized as an important component of Ca flux through these ecosystems. In low-Ca forest soils it was demonstrated that CaOx contribution to the total Ca pool was greater and the turnover rate of CaOx was faster compared to a high-Ca forest ecosystem (Dauer and Perakis 2014). This highlights the importance of CaOx and oxalotrophy in nutrient cycling of dystrophic ecosystems (Dauer and Perakis 2014, Turpault et al. 2019).

The importance of biodiversity and the ecosystem health of the CaOx biogeochemical cycle has only recently been recognized. Soil detritivores such as earthworms, springtails, and termites carry CaOx-degrading bacteria in their digestive systems ​(Cromack et al. 1977, Suryavanshi et al. 2016). Substantial quantities of CaOx from plant litter are processed and ingested by soil fauna such as earthworms and oribatids in deciduous forest ecosystems (Cornaby et al. 1975, Rehman et al. 2021) and, in semi-arid regions, oribatids, and termites play an important role in this process (Francis and Poch 2019) with the litter-transforming oribatids making CaOx-rich litter accessible for subsequent microbial degradation and mineralization (Cornaby et al. 1975, Francis and Poch 2019, Rehman et al. 2021). Work on intact west African forests and adjacent degraded lands has shown that both an intact, healthy, and functioning ecosystem with soil fauna and intact pore spaces are needed for the processes of Ca bioaccumulation to take place (Rehman et al. 2021). Arthropods, diplopods, isopods, oribatids, and snails accumulate significant concentrations of Ca in the form of oxalate and/or carbonate in their exoskeletons (Cromack et al. 1977, Norton and Behan-Pelletier 1991). Unlike CaOx associated with root exudates, fungi, and bacteria, the higher mobility of the soil mesofauna further disperses CaOx throughout soil ecosystems, but this has not yet been factored into soil CaOx budgets.

### Carbon sequestration and the oxalate-carbonate pathway (Goal 13: Climate action, Goal 15: life on land)

Carbon sequestration in both biomass and soils is the major driver of atmospheric CO_2_ drawdown in nature-based solutions, yet the permanence of organic carbon soil sequestration is not always certain (Dees et al. 2023). However, conversion of organic carbon to soil inorganic carbon via the oxalate-carbonate pathway can result in almost permanent carbon sequestration, either in the form of soil carbonates in semi-arid and drier climates or leaching of bicarbonate to groundwater and transport to the ocean in more humid climates, where it counteracts ocean acidification and stores atmospheric carbon (Hartmann et al. 2013, Monger et al. 2015, Taylor et al. 2016, Zhang et al. 2022).

The importance of the oxalate-carbonate pathway in landscape carbon dynamics has been increasingly recognized over the past decade (Cailleau et al. 2011, Martin et al. 2012, Aragno and Verrecchia 2012, Rowley et al. 2017, Pons et al. 2018, Uren 2018, Francis and Poch 2019, Hervé et al. 2021, Rehman et al. 2021). The metabolic breakdown of CaOx by microorganisms produces energy, which is utilized by the organism, as well as inorganic carbon (bicarbonate) and calcium (Uren 2018). Oxidation of CaOx by oxalotrophic microbes may induce a pH shift in soil solution due to the transformation of a stronger acid (oxalic acid; pk1 = 1.25, pk2 = 4.27) to a weaker acid (carbonic acid; pk1 = 6.35, pk2 = 10.33) and concomitant consumption of protons. If the soil environment is sufficiently alkaline, precipitation of calcium carbonate is favored (Braissant et al. 2004, Parsons et al. 2022). An increase of 2.5 pH units was observed in sterilized soils treated with oxalate and oxalotrophic fungi and bacteria after 90 days of incubation, and this shift occurred within <10 days in natural soils harboring an active guild of autochthonous oxalotrophic microorganisms (Martin et al. 2012).

The individual reactions associated with the oxalate-carbonate pathway can be summarised as follows:

Sparingly soluble calcium oxalate dissolves and dissociates:

CaC_2_O_4_ → Ca^2+^ + C_2_O_4_^2−^

 Microorganisms catalyze the aerobic oxidation of oxalate:

2C_2_O_4_^2−^ + O_2_ + 4H^+^ → 2H_2_CO_3_ + 2CO_2_

Because pH increases (protons are consumed) when oxalate is oxidized, carbonic acid will dissociate to form bicarbonate:

H_2_CO_3_ → H^+^ + HCO_3_^−^

And the bicarbonate will dissociate to form carbonate ions:

HCO_3_^−^ → H^+^ + CO_3_^2−^

The calcium released from Ca-oxalate dissolution and the bicarbonate produced from carbon oxidation react to form CaCO_3_:

Ca^2+^ + CO_3_^2−^ → CaCO_3_

The overall reaction can be written in many ways, for example:

2CaC_2_O_4_ + O_2_ + 2H^+^ + 2HCO_3_^−^ → 2CaCO_3_ + 2H_2_O + 4CO_2_,

which shows that 4 moles of carbon in CaOx are oxidized to 4 moles of CO_2_ while the pH shift results in two moles of carbon (as dissolved inorganic carbon) being precipitated as calcite. This representation demonstrates that not all the carbon involved in the OCP is necessarily from oxalate oxidation; ambient bicarbonate derived from “background” soil respiration might contribute to the carbon sequestered as calcite. If we represent all valence four carbons as CO_2_, then reaction six simplifies to:

2CaC_2_O_4_ + O_2_ → 2CaCO_3_ + 2CO_2_

The overall RQ for the OCP process is two.

The Ca and carbon mass balance of an oxalogenic-oxalotrophic ecosystem [soil under a *Milicia excelsa* (Iroko) tree] was modelled according to biogeochemical changes associated with the oxalate-carbonate pathway and 800 kg of calcium carbonate was predicted to accumulate over the course of 170 years in the soil (Gatz-Miller et al. 2022). Approximately 1000 kg of inorganic carbon was predicted to accumulate in soil surrounding an 80-year-old Iroko tree via the oxalate-carbonate pathway (Braissant et al. 2004). Cactus biomass accumulates ∼1.8 × 10^11^ g year^−1^ atmospheric carbon as CaOx across the Sonoran, Chihuahua, Great Basin, and Mojave deserts. The saguaro cactus of Arizona sequesters up to 40 g atmospheric C m^−2^ in CaOx and releases an estimated 2.4 g calcite m^−2^ year^−1^ into the soil (Garvie 2006). Oxalotrophic microorganisms therefore contribute to long-term storage of carbon in mineral form as calcium carbonate, which has a much longer mean residence time in soils compared to organic compounds (Braissant et al. 2004, Verrecchia et al. 2006).

## Climate change and preservation of biodiversity

Nature-based solutions are increasingly recognized as an important tool for harnessing ecosystem services to fight climate change while supporting biodiversity (Seddon et al. 2020). Ecosystems supporting CaOx-rich vegetation in both arid and humid regions potentially provide important carbon sequestration services via the oxalate-carbonate pathway (Garvie 2006, Cailleau et al. 2011, Rehman et al. 2021). Drylands have lower soil organic carbon contents than their more humid counterparts (Lal et al. 2021, Heckman et al. 2023) and so have not been prioritized for conservation, despite occupying ∼40% of Earth's land surface (Sörensen 2007). Oxalotrophy as an ecosystem service for carbon capture in arid and semi-arid regions, as well as more humid regions, is a vital priority for future research. With semi-arid and drier environments occupying almost half the earth's land surface, even small amounts of annual carbon sequestration can have a large impact on atmospheric CO_2_ drawdown (Lal et al. 2021) and the impact of leached bicarbonate generated in tropical ecosystems has not been accounted for, in spite of the fact that the oxalate-carbonate pathway has been documented as important for carbon cycling in west African (Cailleau et al. 2011, Rehman et al. 2021), Amazonian (Cailleau et al. 2014), Indian (Hervé et al. 2018), and Madagascan forest ecosystems (Hervé et al. 2021).

There are also likely to be unique ecosystems where the rates of oxalatrophy are surprisingly high. One such example may be the Subtropical Thicket of South Africa, which is characterized by a closed-canopy vegetation rich in succulent species (Vlok et al. 2003), but also occurs in a semi-arid climate. Given the erratic nature of rainfall in the region, “alarm” photosynthesis, a drought adaptation in plants where CaOx provides a source of CO_2_ during periods of extended stomatal closure (Tooulakou et al. 2016), is likely to be common. More importantly, this type of photosynthesis has been recorded in *Portulacaria afra* (Tooulakou et al. 2016), a stem and leaf succulent tree, that is commonly a dominant component of Subtropical Thicket vegetation (Penzhorn et al. 1974; Vlok et al. 2003). This species promotes litter production, water and sediment trapping, and carbon accumulation (Lechmere-Oertel et al. 2005, 2008, Mills and Cowling 2010; van Luijk et al. 2013) and may be a source of extensive oxalatrophy.

However, in line with the global trends in biodiversity loss (PBES 2019, Pimm and Raven 2019), ecosystems where oxalotrophy is likely, or has been documented, are also being removed at unprecedented rates. Forests in western Africa have decreased by 83% in under 120 years (Aleman et al. 2018). The Amazon is fast approaching the critical threshold of rainforest decline, with 75% of it having lost resilience in the last 20 years (Boulton et al. 2022). Madagascar has lost 25% of its tree cover in the last 22 years (Suzzi-Simmons 2023). Understanding these organic–inorganic carbon biogeochemical processes and recognition of their economic value as natural capital (Dasgupta 2021) are vital for the future management, rehabilitation, and protection of these ecosystems and the ecosystem services they provide.

Because inorganic carbon is stored over a much longer time-frame than organic carbon, nature-based solutions, with a value multiplier such as organic–inorganic carbon transformation, offer increased land value compared to a more transient organic carbon storage. Nature-based solutions with the potential to store carbon for decades are predicted to accrue a cost in the $15–$40 per ton range, whereas engineered solutions with more permanent removal are currently in excess of $200 per ton (and likely will stay above $100 per ton even with technological breakthroughs: WEF 2022). The oxalate-carbonate pathway therefore represents a low cost, nature-based solution that has the carbon storage potential of an engineered solution, and so ecosystems where this takes place offer potential value in terms of their natural capital value (Dasgupta 2021) and this needs to be taken into consideration for ecosystem conservation and regeneration.

## Conclusions

Ecosystems are being lost at an alarming rate without appreciation of the substantial impact that oxalate and oxalotrophy have on ecosystem service provision. Coupled with our lack of understanding of the nonlinear impacts of the removal of such ecosystems, the scale of this destruction may result in magnified negative impacts on human health and well-being, which are directly tied to planetary health and well-being. The role and importance of oxalotrophy in cultivated lands and other ecosystems needs urgent attention to determine how land management practices might influence or capitalize on this carbon capture process. Understanding the intricacies of these organic–inorganic carbon biogeochemical processes is paramount for effectively managing, rehabilitating, and protecting these ecosystems and the ecosystem services they offer.

## Data Availability

There are no new data associated with this manuscript
