# Correction to: Oxalate and oxalotrophy: an environmental perspective

**DOI:** 10.1093/sumbio/qvae004

**Published:** 2024-02-27

This is a correction to: Don A Cowan, Darya Babenko, Ryan Bird, Alf Botha, Daniel O Breecker, Cathy E Clarke, Michele L Francis, Tim Gallagher, Pedro H Lebre, Teneille Nel, Alastair J Potts, Marla Trindade, Lonnie Van Zyl, Oxalate and oxalotrophy: an environmental perspective, Sustainable Microbiology, Volume 1, Issue 1, January 2024, qvad004, https://doi.org/10.1093/sumbio/qvad004.

In the originally published version of this article, typographical errors were present in the names of authors Darya Babenko and Michele L Francis.

This has been corrected.

